# High-density lipoproteins may play a crucial role in COVID-19

**DOI:** 10.1186/s12985-022-01865-4

**Published:** 2022-08-23

**Authors:** Minu Nain, Apoorv Gupta, Sumit Malhotra, Amit Sharma

**Affiliations:** 1 National Institute of Malaria Research, New Delhi, 110 077 India; 2 Centre for Community Medicine, All India Institute of Medical Sciences, New Delhi, 110029 India; 3 Molecular Medicine, International Centre for Genetic Engineering and Biotechnology, Aruna Asaf Ali Marg, New Delhi, 110 067 India

**Keywords:** Lipids, HDL, Cholesterol, Viruses, COVID-19

## Abstract

**Background:**

Lipids play a central role in the virus life cycle and are a crucial target to develop antiviral therapeutics. Importantly, among the other lipoproteins, the ‘good cholesterol’ high-density lipoprotein (HDL) has been widely studied for its role in not only cardiovascular but several infectious diseases as well. Studies have suggested a role of serum lipids and lipoproteins including HDL, total cholesterol (TC), triglycerides (TG), and low-density lipoproteins (LDL) in several viral infections including COVID-19. This disease is currently a major public health problem and there is a need to explore the role of these host lipids/lipoproteins in virus pathogenesis.

**Methodology:**

A total of 75 retrospective COVID-19 positive serum samples and 10 COVID-19 negative controls were studied for their lipid profiles including TC, HDL, LDL, and very-low-density lipoproteins (VLDL), and TG.

**Results:**

Systematic literature search on dyslipidemia status in India shows that low HDL is the most common dyslipidemia. In this cohort, 65% (49) of COVID-19 patients had severely low HDL levels whereas 35% (26) had moderately low HDL and none had normal HDL levels. On the other hand, ~ 96% of samples had normal TC (72) and LDL (72) levels. VLDL and TG levels were also variable. In the controls, 100% of samples had moderately low HDL but none severely low HDL levels.

**Conclusion:**

HDL likely plays a crucial role in COVID-19 infection and outcomes. The causal relationships between HDL levels and COVID-19 need to be studied extensively for an understanding of disease pathogenesis and management.

**Supplementary Information:**

The online version contains supplementary material available at 10.1186/s12985-022-01865-4.

## Introduction

Lipoproteins are complex particles composed of cholesterol esters, free cholesterol, phospholipids, apolipoproteins, and triglycerides [1]. They act as lipid carriers and are categorized into seven classes i.e. low-density lipoproteins (LDL), very low-density lipoproteins (VLDL), high-density lipoproteins (HDL), chylomicrons, chylomicrons remnants, intermediate-density lipoproteins (IDL) and lipoprotein a (Lpa) [1]. Amongst these, only HDL is anti-atherogenic while all others are pro-atherogenic [2, 3]. HDL are heterogeneous lipid carriers composed of lipids, proteins, enzymes, bile acids, and miRNAs [4]. The liver is the central organ for HDL synthesis as well as recycling and recirculation of lipid-rich HDL particles. Despite the dynamic composition of HDL, cholesterol is still the most widely used clinical biomarker for HDL [5]. HDL is responsible for reverse cholesterol transport (RCT) where it transports excessive cholesterol to the liver thus preventing its accumulation in peripheral arteries [5]. Apart from RCT, HDL protects LDL from free radical oxidation thus preventing the accumulation of oxidation products in the arterial wall [3, 6]. These functions of HDL make it a ‘good cholesterol’. HDL is distributed in different subclasses based on their size, density, protein, lipid composition, and electrophoretic mobility [7].

Apart from playing a crucial role as the structural component of cells, lipids also act as signaling mediators [8, 9], energy resources, and conduct antiviral host immune responses [10, 11]. Altered plasma lipid levels have been earlier noted in multiple viruses including Human immunodeficiency virus (HIV), Hepatitis C, Dengue, and Epstein-Barr virus (EBV) [12–14]. Dengue virus modulates host lipid metabolism for its replication [15]. Total cholesterol (TC), LDL, and HDL were inversely correlated to dengue severity [15] and HDL levels were found to be reduced during infection. The apoA-1, an important component of HDL has been shown to render antiviral properties and apart from inhibiting DENV entry into host cells, it prevents cell penetration of herpes and HIV by inhibiting fusion with the host cell membrane [16]. Dyslipidemia, more importantly, reduced HDL levels are the most common abnormality reported in 85–90% of antiretroviral therapy (ART) naïve and treated HIV patients [17, 18]. CVD following HIV infection is frequently reported mainly due to changes in composition and levels of HDL and LDL during HIV infection [19]. Lipids play an important role in the Hepatitis C virus life cycle including entry, replication, packaging, and egress [20, 21]. The apoM, another important HDL component is enhanced during HBV infection and seems to suppress its replication [22]. EBV infection is also shown to be associated with atherosclerotic risk. In acute EBV infections, the levels of TC, LDL, HDL, apoA-1, apoB-1 are reduced and TG is increased [14]. In most of these viral infections, there are infection-induced alterations in lipid metabolism and serum lipid, lipoproteins, and associated enzyme levels, and abnormal HDL levels are shown to be associated with the risk of developing several infectious diseases [23, 24].

As observed with other viral infections, there have been multiple reports of changes in lipid profile levels in COVID-19 patients [25, 26] since the beginning of the COVID-19 pandemic. These are marked by a decrease in total cholesterol, LDL, HDL, and variable TG levels [27–29]. Decreased levels of TC, HDL, LDL and increased TG are also shown to be positively correlated with disease severity [30, 31]. It was observed that TC, HDL, and LDL kept declining as the disease progresses and starts getting normal/base level as the clinical symptoms improve [27, 32]. The decreased levels of HDL, LDL, and increased levels of TG may be predictors for disease severity but not mortality [33]. The study on lipid profiles of ICU patients showed decreased levels of LDL and HDL levels, supporting the role of these lipoproteins in COVID-19 pathogenesis [34]. This change of lipid profile in COVID-19 patients increases the risk for CVD in these patients by altering cholesterol metabolism at the cellular level and hence altering the serum lipid levels [35]. Thus, COVID-19 induced heart failure can be attributed to the complex interplay between dysregulation of lipid levels, cytokine storm, and multiple neuro-metabolic derangements [36]. There is no conclusive report on the exact mechanism of HDL reduction during COVID-19 to date and certainly needs to be explored thoroughly. Nevertheless, there are a few probable explanations for the same including the role of HDL in SARS-CoV-2 binding and entry, which might lead to HDL consumption and hence reduced HDL levels during COVID-19 [37]. Also, HDL isolated from COVID-19 patients shows a protective effect against TNF-alpha–induced increase in vascular endothelial–cadherin disorganization, apoptosis, and endothelial cell permeability [38]. Hence, HDL can incur a protective effect against COVID-19 via modulating inflammatory response [39] as well as due to its antioxidant, cytopathic, and anti-apoptotic properties [40, 41].

Apart from serum HDL levels, changes in HDL composition and function affect COVID-19 pathogenesis and outcome [42]. HDL from COVID-19 individuals with decreased levels of Apo A-1 and paraoxonase 1(PON) proteins shows compromised anti-apoptotic activity as compared to native HDL from non-COVID-19 controls [38]. In the elderly and comorbid individuals, there is oxidation and glycation of HDL resulting in reduced antiviral and anti-inflammatory activity [39]. This pro-atherogenic and pro-inflammatory activity of glycated HDL might explain the COVID-19 severity in elderly and co-morbid individuals [39]. This significant variation in HDL composition between mild, moderate, and severe COVID-19 infections, indicates the role of HDL in COVID-19-induced hyperinflammation [38, 42]. Changes in protein and lipid composition of HDL particles upon viral infection may transform the anti-inflammatory HDL to pro-inflammatory HDL particles which are less protective in endothelial cells and might promote TNFα and MCP-1 production by macrophages/monocytes [43–45]. These pro-inflammatory HDLs might lead to endothelial injury contributing to COVID-19-associated hyper inflammation and organ failure [38]. Sphingosine-1 phosphate(S1P) is an important component of HDL and facilitates endothelial permeability [46, 47]. It also exerts anti-apoptotic and anti-inflammatory effects by interacting with the sphingosine-1 phosphate receptor [48]. S1P dampens the cytokine storm by inhibiting the release of pro-inflammatory cytokines [49, 50]. So, modified S1P levels as a result of alterations in HDL levels increases the chances of disrupted endothelial cell structure and function as well as an enhanced proinflammatory responses during COVID-19 [51]. S1PR modulators like fingolimod, Siponimod and ozanimod are currently being explored in COVID-19 treatment [50]. Also, HDL prevents ROS synthesis and averts LDL oxidation as well as copper ion-induced oxidative damage [52]. ApaA1, S1P, ApoE, ApoM, and hydrolases like PON, and lecithin-cholesterol acyltransferase(LCAT) are the HDL components with anti-oxidative properties [53]. Thus, both HDL levels and composition may alter its anti-oxidative properties even during COVID-19.

Dyslipidemia is quite common in the world including in India as is evident from studies in the last few decades. As per these reports, low HDL is the most common dyslipidemia (Table 1). Given the crucial role of lipids and lipoproteins in the pathogen/virus life cycle, the high prevalence of dyslipidemia puts more than half of the population at the risk of developing diseases including COVID-19 and CVD. Hence there is a need to study the role of these lipids in COVID-19 infections. Since its first report in Wuhan in Dec 2020, it has claimed 5,757,562 deaths worldwide [54]. People with comorbidities like hypertension, obesity, and diabetes mellitus are more prone to developing severe disease and mortality [55, 56]

**Table 1:** Prevalence (%) of abnormal total cholesterol, high-density lipoproteins, low-density lipoproteins, and triglyceride levels in the Indian population as documented in several studies across different parts of the country

Authors/references	No of subjects	Pubmed Id	Year	High TC (%) > 200 mg/dl	High LDL (%) > 130 mg/dl	Low HDL (%) < 55 mg/dl	TG (%) > 150 mg/dl
Gupta et al. [60]	1800	11,999,090	2002	39.1	41.5	55	–
Prabhakaran et al. [59]	2122	15,981,439	2005	30.1	67.2	33	–
Mehan et al. [62]	220	16,855,315	2006	40.5	–	–	–
Gupta et al. [57]	1091	18,051,732	2007	39.1	41.5	55	–
Chow et al. [58]	345	16,839,628	2007	12.3	12.3	87.2	–
Kaur et al. [61]	2262	18,290,552	2007	30.3	–	–	–
Sawant et al. [75]	1805	18,472,509	2008	38.7	–	64.2	–
Sawant et al. [75]	1805	18,472,509	2008	23.3	–	33.8	–
Choa et al. (Male data) [63]	1375	20,876,149	2010	–	–	–	26.9
Choa et al. (Female data) [63]	608	20,876,149	2010	–	–	–	27.4
Pandey et al. [64]	4435	21,880,382	2013	–	–	–	20
Joshi et al. [66]	2000	24,817,067	2014	13.9	11.8	72.3	29.5
Guptha et al. (Male data) [65]	3388	24,973,832	2014	25.1	16.3	33.6	–
Guptha et al. (Female data) [65]	2735	24,973,832	2014	25.1	16.3	33.6	–
Ajay Raj et al. (Urban) [76]	325	–	2016	30.9	33.4	30.9	47.9
Ajay Raj et al. (Rural) [76]	325	–	2016	25.1	23.1	36.9	40.0
Gupta et al. (Male data) [67]	2102	28,648,438	2017	–	–	–	32.2
Gupta et al. (Female data) [67]	781	28,648,438	2017	–	–	–	20.1
Gupta et al. [77]	50	–	2018	2	4	4	14
Fatmi et al. (Chennai, India) [78]	6906	33,904,416	2021	31.3	29.1	49.7	34.6
Fatmi et al. (Delhi, India) [78]	5364	33,904,416	2021	28.8	21.2	67.1	33.6

To study the role of lipoproteins in COVID-19 infection, we aimed to assess the lipid profile in COVID-19 positive serum samples from Delhi, India. These lipid profile studies will help fill the gap in our understanding of disease pathogenesis and may pave the way for more elaborate molecular and clinical studies on the role of lipids/lipoproteins in the virus life cycle.

## Methodology

### Dyslipidemia data India

The terms “Dyslipidemia”, “prevalence” and “hypercholesterolemia” were used to search the registered and published studies in PubMed, and Google Scholar with filters “only humans source”, “Indian studies” and the year “2000 onwards”. After removing duplication, manual screening of retrieved Indian studies was based on abstracts and titles for their potential inclusion. In the screening studies, those that have at least one of the lipid profile parameters i.e. HDL, LDL, TC, VLDL, and TG studied in the population were retained. In addition, full texts of the included studies were reviewed for eligibility, and those with missing information on prevalence data were excluded from the final dataset. The data was extracted from the final dataset based on “Author”, “No of subjects”, “PubMed ID”, “Year”, “HDL prevalence (%)”, “LDL prevalence (%)”, “Cholesterol Prevalence (%)”, “Triglycerides prevalence (%)”.

### Sample selection

5–10 ml blood samples from COVID-19-positive patients, as confirmed by RT-PCR of nasal/throat swab were collected under the Indian Council of Medical Research (ICMR)’s project ‘Establishment of a network of ICMR-COVID-19 biorepositories in India’. Under this, bio-repository of duly specified clinical samples of COVID-19 patients are maintained at individual institutions for research to better understand COVID-19 disease and develop novel solutions for COVID-19 prevention, control, and treatment. For this study, 75 retrospective samples from the biorepository were tested for their lipid profiles. Also, blood samples of 10 healthy volunteers with no history of COVID-19 infection were collected after consent.

### Serum isolation and lipid profile test

The whole blood from COVID-19 positive patients was collected in vacutainers and kept undisturbed at room temperature for 30 min followed by centrifugation at 1000–2000 g for 10 min in a refrigerated centrifuge. The resulting supernatant was the serum which was then carefully transferred to polypropylene and processed for lipid profile testing. The remaining serum is aliquoted and stored at − 80 °C for long-term use.

Lipid profiles including triglyceride, HDL cholesterol, total cholesterol, VLDL cholesterol, LDL cholesterol, total/HDL cholesterol ratio, LDL/HDL cholesterol ratio of 75 COVID-19 positive serum samples and 10 COVID-19 negative samples were tested by standard enzymatic, colorimetric assays. The normal ranges for these lipids are summarized in Additional file 1: Table S1.

### Limitations of the study

This study includes mild/moderate patient samples from a single geographical region and a single time point. As the samples were retrospective and COVID-19 was widespread with asymptomatic and multiple infections, only 10 healthy negative controls without COVID-19 infection ever, could be enrolled for the current study. Also, the age data of only 66/75 tested samples are available. The role of lipid dysregulation in COVID-19 severity, susceptibility, pathogenesis, and outcome need to be explored in different cohorts based on disease symptoms/severity, co-morbidities, and disease progression.

## Results

### Status of dyslipidemia in India

**Fig. 1:**
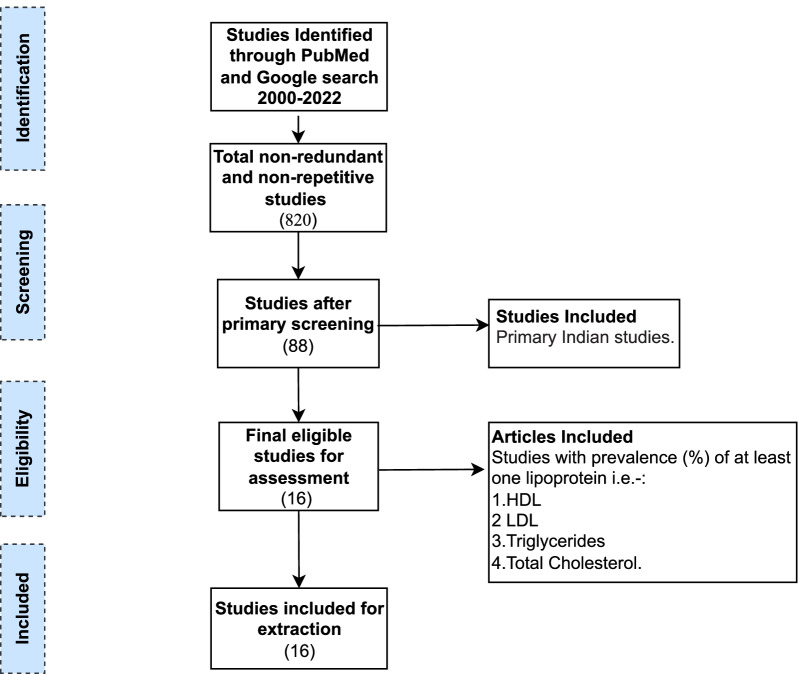
Consort-flow diagram to identify literature on the “Status of dyslipidemia in India” in the last two decades

The comprehensive search yielded 820 de-duplicated studies and after subjection to the screening, the final dataset contained 15 articles from the years 2006–2021 with prevalence (%) of different lipoproteins. The data extracted and structure have been summarized in Table 1, Fig. 1. The data show that moderate to severe low HDL is prevalent in 33–87% Indian population whereas 12–67% and 13–40% of individuals have high LDL and high TC levels respectively (Table 1) [57–67]. The variation in the prevalence rate of different lipoproteins is attributed to the socioeconomic status of the study population, diversity of selected study sites, and sample size. However, the latest data available from the study by the Indian Council of Medical Research-India Diabetes with diverse study sites shows the prevalence of low HDL is as high as 72.3% of tested individuals while high TC and LDL were found in 13.9% and 11.8% individuals. Low HDL is the most common dyslipidemia found in all these studies.

### Levels and distribution of lipids in COVID-19 positive patients

**Fig. 2:**
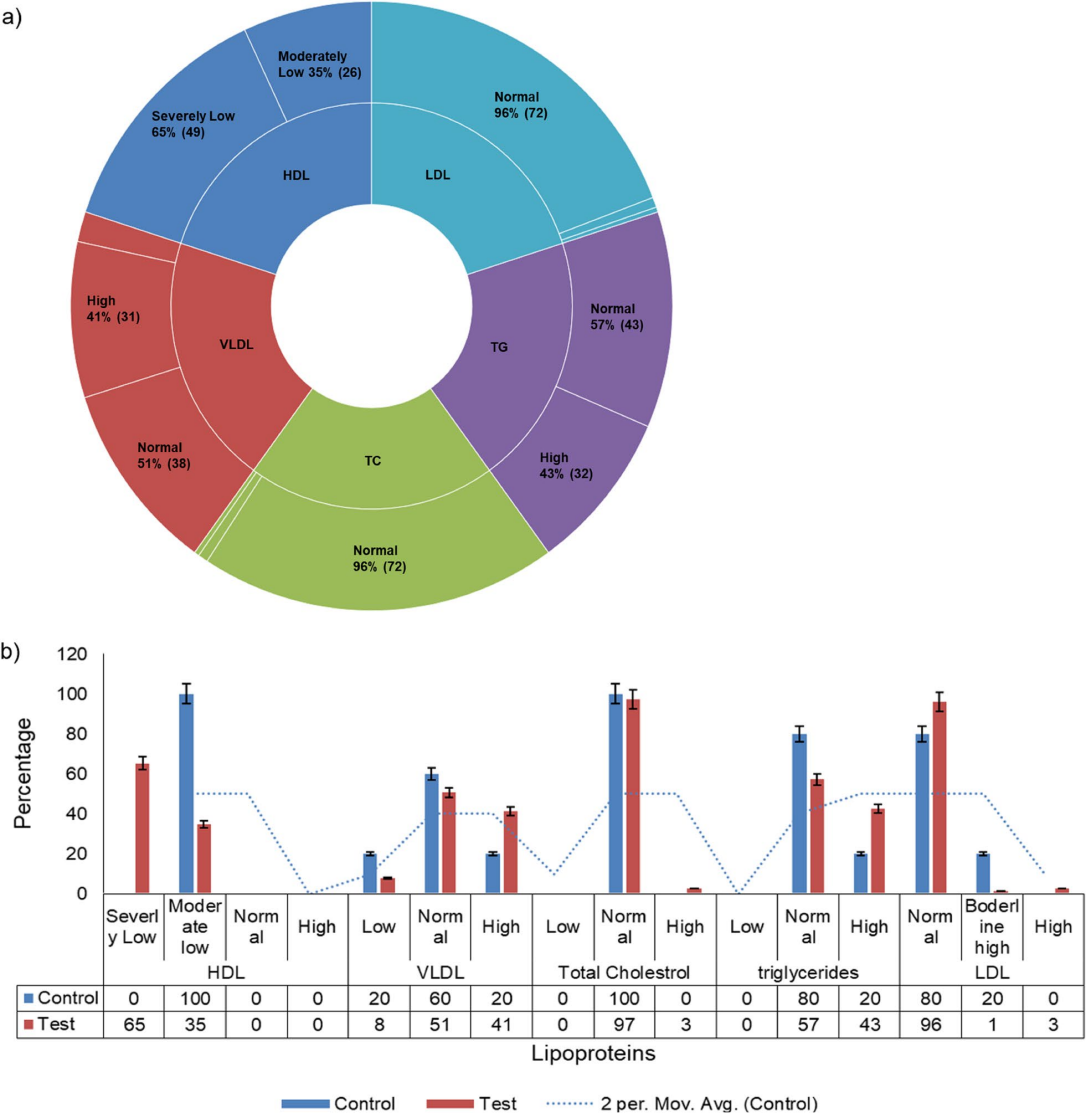
**a** Lipid status of COVID-19 patients. The levels and distribution of lipoproteins in 75 COVID-19 positive patient serum samples. HDL levels are reduced, LDL and TC remain normal whereas most of the individuals have normal or increased TG and VLDL levels. **b** Test versus control. Lipid profile prevalence (%) in COVID-19 positive and control samples

**Fig. 3:**
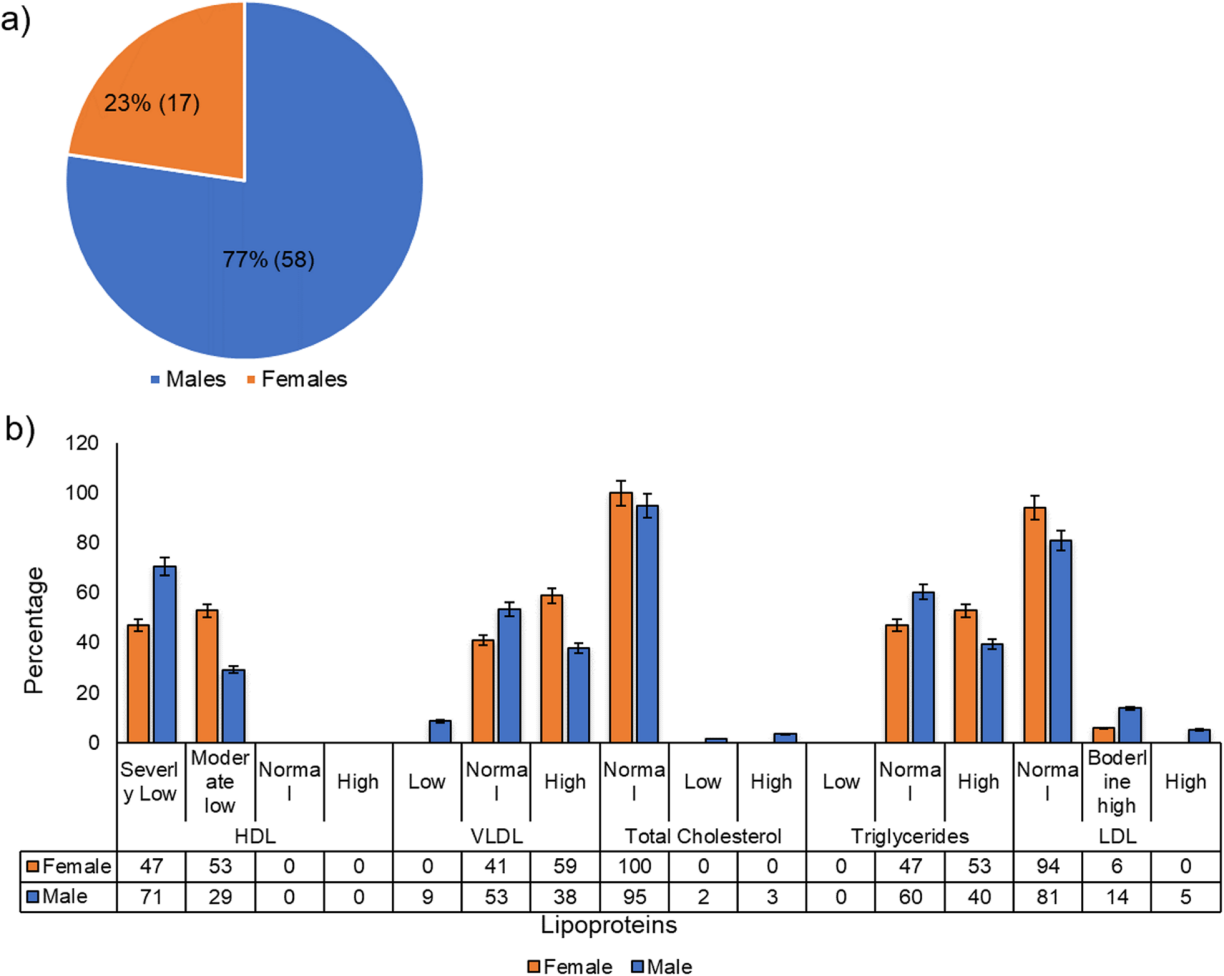
**a**, **b** Levels and distribution of lipids in Males (58/75), and females (17/75)

65% of COVID-19 patients had severely low HDL levels whereas 35% had moderately low HDL and none had normal HDL levels (Fig. 2a). The majority of males fall under the severely low HDL category (Fig. 3a, b). In the case of total cholesterol, only 3% of test samples had high TC, and the rest 96% had normal TC while only 1 patient had TC in the low range. All females tested had normal TC levels and 5% of males had low or high TC (Fig. 3a, b). Similarly, LDL levels seemed to be unperturbed in the case of COVID-19 infection here unlike other published studies as 96% had normal, whereas only 1% and 2% had moderate levels and very high LDL levels respectively. (Fig. 2a). The distribution levels of LDL seem independent of genders (Fig. 3a, b).

The levels of TG and VLDL were however variable among the tested samples. Upto 43% had high TG levels, 57% had normal while none had low TG. For VLDL, 41% had elevated, 8% had below normal, and the rest 51% had VLDL in the normal range (Fig. 2a). There was no significant difference in TG and VLDL levels between males and females (Fig. 3a, b).

**Fig. 4:**
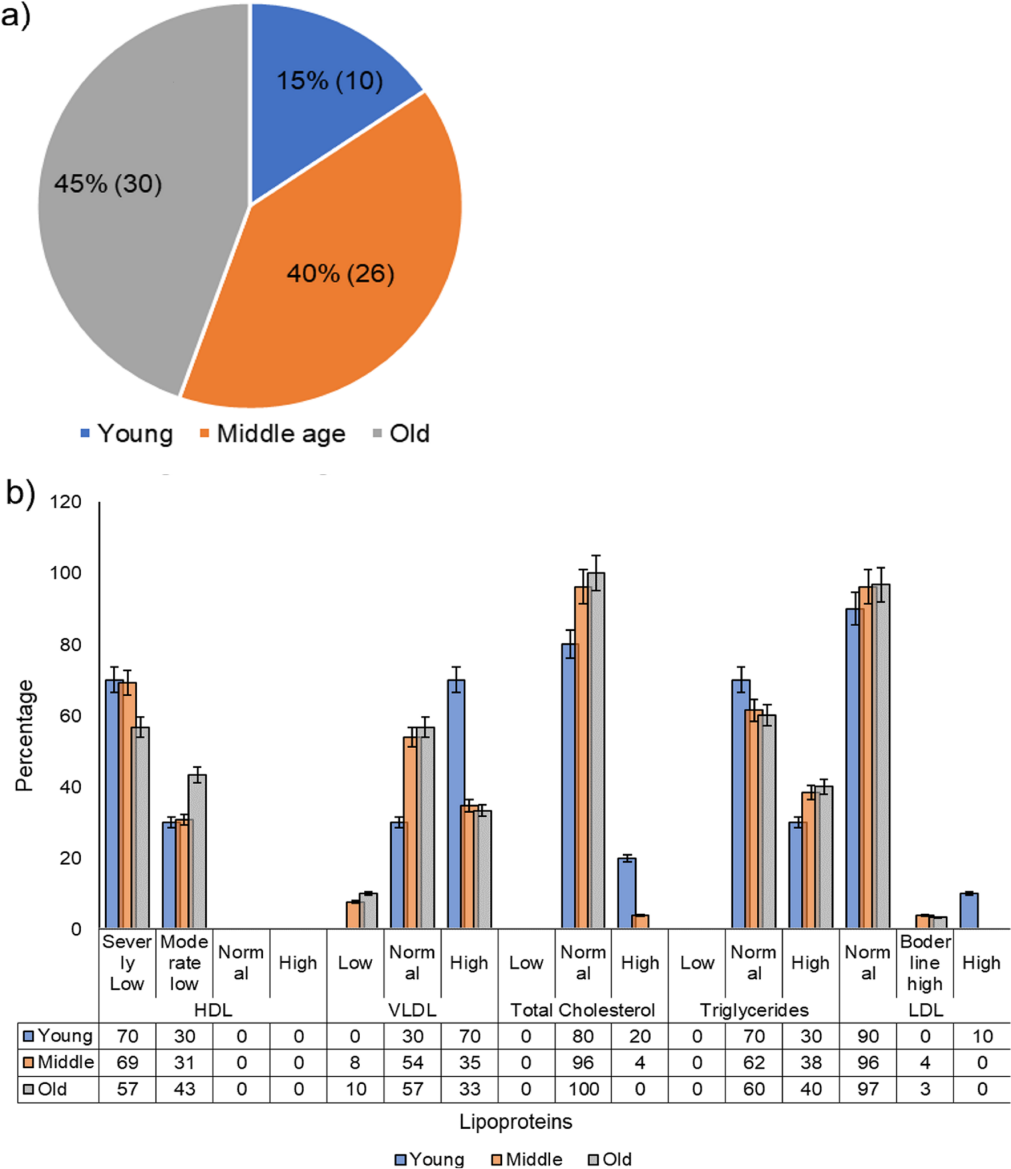
**a**, **b** Levels and distribution of lipids in different age groups, young, < 40Y (10/66), middle, 40–60 Y (30/66), and old, > 60Y (26/66)

Among the control samples, 100% samples had normal TC, moderately low HDL, and none with severely low HDL levels, unlike test samples where 65% samples had HDL in the severely low category (Fig. 2b). This shows that HDL might play a crucial role in either susceptibility to COVID-19 infection or disease pathogenesis. More elaborate studies are needed to study the HDL and COVID-19 causal relationship. Upto 80% of control samples had normal TG as compared to 57% of test samples (Fig. 2b). LDL levels were normal in 80% of control and 96% of test groups (Fig. 2b). The difference between the lipid profile of the test and control samples was however inconclusive due to the very small sample size of the control group. TC was normal in 100% of old age group > 60Y whereas 20% of young < 40Y individuals had low TC and 80% had normal TC (Fig. 4a, b). There was no significant difference in distribution pattern and lipid levels of other lipoproteins among different age groups (Fig. 4a, b). Lipid profiles in comorbid individuals show almost the same lipid profile as in non-comorbid individuals. These data suggest that the levels of HDL were reduced whereas that of LDL remained unperturbed during COVID-19 infection. TG and VLDL levels remained normal or elevated in most of the tested samples.

## Discussion

The crucial role of lipids and lipoproteins in the virus life cycle makes them a promising target for developing therapeutics against infectious diseases. Given the significant role of these lipids in the virus life cycle, dyslipidemia not only elevates the risk of CVD but also along with other demographic and clinical factors might put people at the risk of developing several viral infections like Dengue, hepatitis C, HIV, EBV and COVID-19. Thus, interventions targeting lipids and lipoproteins can play a significant role in combating infections, especially when viral. As per the latest dyslipidemia statement by the Lipid Association of India Expert Consensus, ~ 79% of Indians have at least one lipid abnormality, with decreased HDL in 72.3% of individuals, hypertriglyceridemia in 29.5%, and elevated LDL in 11.8% [68]. The high prevalence of dyslipidemia shows that majority of the population might be at risk of developing virus infections including COVID-19 and associated complications. In this study, we evaluated the lipid profile of COVID-19 positive patients to explore the causal relationship between lipids and COVID-19 infection. Earlier studies on the levels of lipids in COVID-19 patients suggested that the levels of TC, HDL, and LDL decreased whereas TG levels increased during infection. In line with this, our study showed that the levels of HDL were low while LDL and TC levels remained normal in the COVID-19 positive samples. The TG and VLDL levels varied more dynamically and remained inconclusive. The distribution and lipid profile levels remained almost the same in samples irrespective of gender, age, and comorbidities with almost all samples having low HDL. The higher risk of developing severe COVID-19 infection and associated mortality in a comorbid population with already abnormal lipid levels suggests a crucial role for these lipids in COVID-19 pathogenesis. Statin, an HDL-raising drug has been previously shown to inhibit replication in several positive-strand RNA viruses including Hepatitis C, Dengue, West Nile, and Influenza A [69, 70]. Disease management by administering such HDL infusion therapies and HDL-raising drugs like statins and finofibrate-Crataegus oxyacantha combination therapy in COVID-19 treatment may be a promising option. To develop HDL-based therapies [71–73], explorations of the causative relationships between HDL levels and infection risk are needed. It is necessary to study and understand whether the low abnormal levels of lipids are risk factors for COVID-19 infection or whether it is the COVID-19 infection that leads to abnormal host lipids. Infection-induced alterations in lipoproteins levels and composition might lead to the development of CVD in the long run. These cases will need further care and follow-ups to assess their cardiovascular health. Physicochemical composition and quality of HDL can be decisive for its harmful or useful effects. HDL can change its composition and shift to another subclass in the presence of risk factors like a cardiovascular or foreign infection [7, 74]. So, there is also a need to shift the focus and identify more potent, accurate, and functionally significant biomarkers for HDL function from other components of HDL particles including proteins, enzymes, and miRNAs. The crucial role of lipids in the virus infection cycle can be utilized to develop broad-spectrum antiviral drugs. These lipid-based drugs, either alone or in combination can be used to target multiple virus infections including COVID-19.

## Supplementary Information

**Additional file 1.** **Supplementary Table 1.** Standard ranges for multiple lipids.

## Data Availability

The datasets generated during and/or analyzed during the current study are available from the corresponding author on reasonable request.
