# Supplementary material for: High-density lipoproteins may play a crucial role in COVID-19
Source: Virol J. 2022 Aug 23;19:135. doi: 10.1186/s12985-022-01865-4 (PMC9395887; doi:10.1186/s12985-022-01865-4)
Supplement: Supplementary file 1 — Additional file 1. Supplementary Table 1. Standard ranges for multiple lipids. [file 12985_2022_1865_MOESM1_ESM.docx]

| **Lipid profile** | **Ranges** | **Range values(µg/dl)** |
| --- | --- | --- |
| Triglyceride | Normal | 0-200 |
| Total Cholesterol | Desirable | <200 |
|  | Borderline high | 201-240 |
|  | High | >240 |
| HDL Cholesterol | Normal | > 55 |
|  | Moderate low | 35-55 |
|  | Severe low | < 35 |
| VLDL Cholesterol | Normal | 15.0-40.0 |
| LDL Cholesterol | Optimal | < 100 |
|  | Near-optimal | 100-129 |
|  | Borderline high | 130-159 |
|  | High | 160-189 |
|  | Very high | >190 |

**Supplementary Table 1.** Standard ranges for multiple lipids.
